# Associations of physical activity domains and muscle strength exercise with non-alcoholic fatty liver disease: a nation-wide cohort study

**DOI:** 10.1038/s41598-023-31686-6

**Published:** 2023-03-23

**Authors:** Yewan Park, Dong Hyun Sinn, Kyunga Kim, Geum-Youn Gwak

**Affiliations:** 1 Department of Internal Medicine, Kyung Hee University Hospital, Seoul, Korea; 2 Department of Digital Health, SAIHST, Sungkyunkwan University, Seoul, Korea; 3 Department of Medicine, Samsung Medical Center, Sungkyunkwan University School of Medicine, Seoul, Korea; 4 Statistics and Data Center, Research Institute for Future Medicine, Samsung Medical Center, Seoul, Korea

**Keywords:** Non-alcoholic fatty liver disease, Risk factors

## Abstract

It is unclear if various types and domains of exercise have an identical effect on non-alcoholic fatty liver disease (NAFLD). Thus, this study aimed to investigate associations of different physical activity domains and muscle strength exercise with NAFLD using a nation-wide cohort database. Adults aged 20–79 years who participated in the Korean National Health and Nutrition Examination Survey between 2014 and 2018 were analyzed. Hepatic steatosis index was used to identify NAFLD. Physical activity was assessed with the Global Physical Activity Questionnaire. Of 21,015 participants, 4942 (23.5%) had NAFLD. Participants with ≥ 150 min/week of total physical activity had a lower risk of NAFLD than those with < 150 min/week (the fully adjusted OR: 0.86, 95% CI 0.78–0.95). When the individual domain of physical activity was assessed, ≥ 150 min/week of recreation activity was associated with a reduced risk of NAFLD (OR: 0.77, 95% CI 0.67–0.88), whereas ≥ 150 min/week of travel or work activity was not. The fully adjusted OR for NAFLD comparing participants with ≥ 2/week to those with < 2/week of muscle strength exercise was 0.83 (95% CI 0.73–0.94). Muscle strength exercise ≥ 2/week showed a lower risk of NAFLD for all levels of total and each specific domains of physical activity except for ≥ 150 min/week of work activity. An increased level of physical activity and muscle strength exercise was associated with a reduced risk of NAFLD, albeit the effect varied depending on domains of physical activity. Thus, physical activity should be differentiated by domains for the management of NAFLD. Muscle strength exercise could also be a good option for individuals who could not perform moderate-to-vigorous physical activity.

## Introduction

Non-alcoholic fatty liver disease (NAFLD) is characterized by the accumulation of fat in the liver without secondary causes^1^. It is closely related to obesity, diabetes, dyslipidemia, and metabolic syndrome^2–4^. NAFLD is one of the most prevalent liver diseases worldwide, with an estimated prevalence of 24%^5^.

Lifestyle modification such as weight reduction through a hypocaloric diet and exercise serves as the basis for the treatment of NAFLD in the absence of pharmacological agents^1,6,7^. The majority of studies suggesting a favorable benefit have focused on recreational physical activity, leaving the kind, intensity, and duration of physical activity necessary for optimal therapeutic outcomes in the management of NAFLD unclear^7,8^. The World Health Organization (WHO) 2020 guideline has stated that moderate-to-vigorous physical activity in any domain is beneficial. Until now, evidence to conclude whether health benefits of physical activity vary by type or domain is insufficient^9^. In addition, the WHO 2020 guideline recommends muscle strength exercise more than twice a week for all adults^9^. However, recent studies have reported contradictory effects of work-related physical activity on health, such as HOMA-IR^10^. Diabetes^11^, blood pressure^12^, coronary heart disease^13,14^, and cardiovascular mortality^14–16^. Likewise, growing evidence indicates that work-related physical activity is not protective against NAFLD^17,18^, prompting further research to investigate whether NAFLD is influenced differently by the domain of physical activity. Also, how muscle strength exercise interacts with physical activity to affect NAFLD remains unclear.

Therefore, the objective of this study was to investigate associations of different physical activity domains and muscle strength exercise with NAFLD using the Korean National Health and Nutrition Examination Survey (KNHANES).

## Materials and methods

### Study design, setting, and participants

The KNHANES is a nation-wide surveillance system to monitor the health and nutritional status of the general population of South Korea^19^. Each year, representative samples of approximately 10,000 people are selected. Health examination, health interview, and nutritional survey are then conducted.

**Figure 1:**
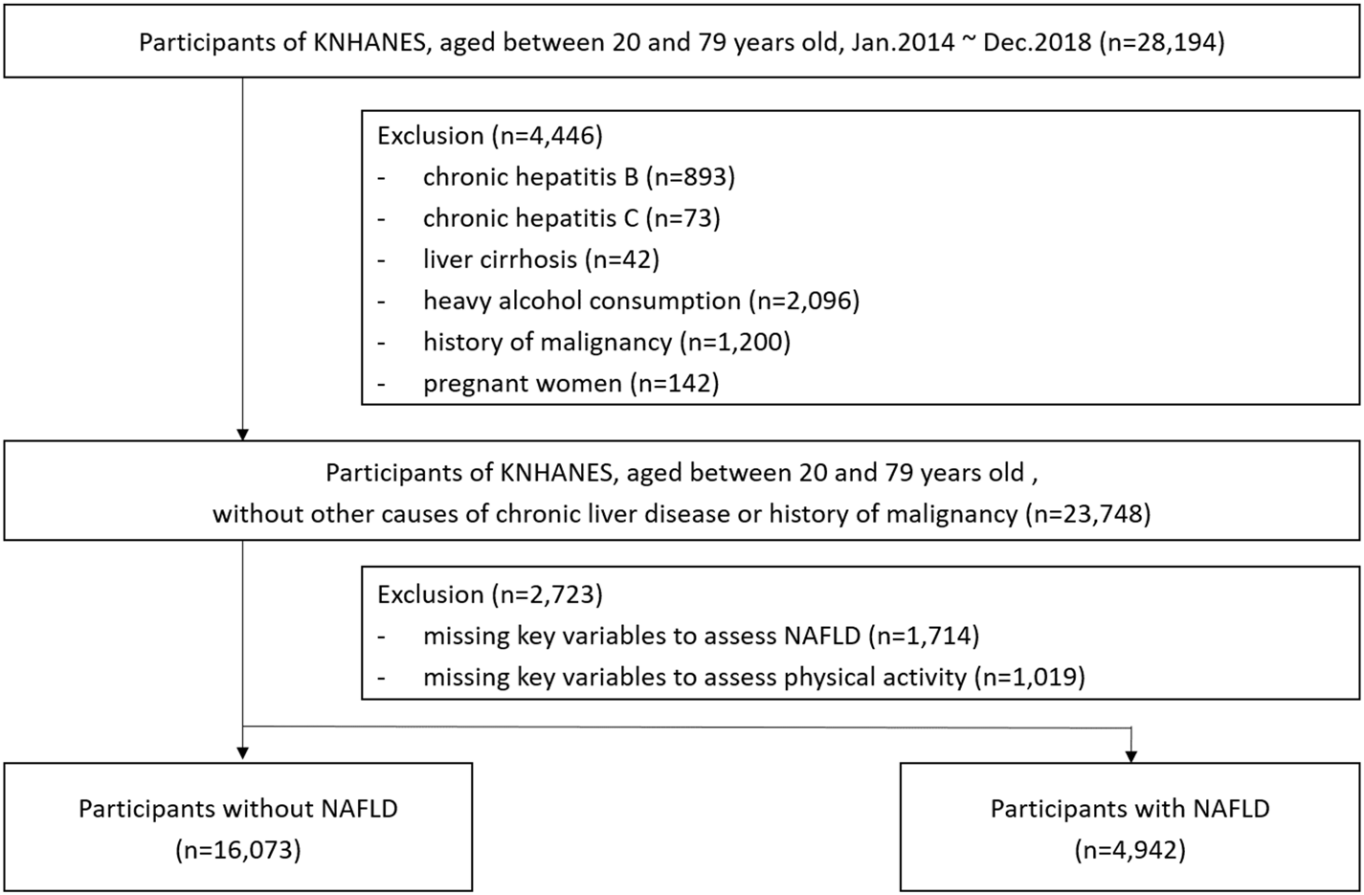
Flowchart showing the selection of study participants.

We screened a total of 28,194 adult men or women aged 20–79 years who participated in the KNHANES from January 2014 to December 2018. Among them, we excluded 4446 participants who met the following exclusion criteria to include participants without chronic viral hepatitis, liver cirrhosis, heavy alcohol use, or malignancy: (1) chronic hepatitis B (n = 893, determined by the presence of hepatitis B surface antigen); (2) chronic hepatitis C (n = 73, determined by the presence of hepatitis C virus RNA test or history of chronic hepatitis C); (3) liver cirrhosis (n = 42, determined by a history of liver cirrhosis); 4) heavy alcohol intake (n = 2096, 30 g or more for a day for men and 20 g or more for a day for women)^6^; (5) history of malignancy (n = 1200); and (6) pregnant women (n = 142). Of these participants, we further excluded 2723 participants missing key variables for assessing NAFLD [n = 1714: missing values for alanine aminotransferase (n = 1126), heights (n = 44), body weights (n = 2), and alcohol intake (n = 542)] or missing key information on physical activity (n = 1019). Finally, a total of 21,025 participants were analyzed (Fig. 1). The survey was conducted after receiving written informed consent from all study participants. The study protocol was reviewed and approved by the Institutional Review Board of the Korea Disease Control and Prevention Agency (No: 2013-12EXP-03-5C, 2018-01-03-P-A) and the Samsung Medical Center (No: 2021-01-013). The study was performed in accordance with the Declaration of Helsinki.

### Study outcomes, variables, and measurements

The diagnosis of NAFLD was made using hepatic steatosis index (HSI)^20^. HSI consists of aspartate aminotransferase, alanine aminotransferase, sex, body mass index (BMI), and diabetes mellitus. Participants with HSI of 36 or higher were considered to have NAFLD. The health interview including physical activity was conducted by trained surveyors consisting of nurses and epidemiologists. To gather comprehensive physical activity information, the Global Physical Activity Questionnaire (GPAQ) and frequency of muscle strength exercise were collected. The level of physical activity was interviewed using the Korean version of GPAQ^21^. The GPAQ was originally developed by WHO to monitor physical activity in numerous countries. It is grouped into three domains of physical activity: recreation, travel, and work activities. The recreation domain includes sports, fitness, and leisure activities. The travel domain includes transport to and from places. The work domain has paid or unpaid work, study/training, household chores, harvesting food/crops, fishing or hunting for food, and seeking employment. The GPAQ provides information on the frequency (times in a week) and duration (minutes at a day) of each domain of physical activity. In recreation and work domains, the intensity of physical activity was also provided (moderate or vigorous). Results were analyzed as suggested by the WHO: (1) the duration of vigorous physical activity was doubled and added to the duration of moderate physical activity, (2) three domains of physical activity were summed to calculate the duration of total physical activity. Since the WHO guideline states that all adults should do at least 150 min/week of moderate-intensity aerobic physical activity, total and each domain of physical activity were divided into < 150 min/week and ≥ 150 min/week. To investigate additional benefits of ≥ 300 min/week of physical activity, we performed further categorization: 0 min/week, 1–149 min/week, 150–299 min/week, and ≥ 300 min/week. The frequency of muscle strength exercise was determined by the number of muscle strength exercise in a week. Since the WHO 2020 guideline recommends that all adults should perform muscle strength exercise at least twice a week^9^, muscle strength exercise was categorized as < 2/week and ≥ 2/week.

Variables collected were age, sex, height, body weight, waist circumference, triglyceride, high density cholesterol, systolic blood pressure, diastolic blood pressure, fasting serum glucose, hepatitis B surface antigen, anti-HCV antibody, history of chronic hepatitis C, history of malignancy, history of liver cirrhosis, use of antihypertensive medications, antidiabetic medications, antidyslipidemic medications, alcohol use behavior, smoking status, pregnancy, household income information, and education level. BMI was calculated as weight in kilograms/height in square meters (kg/m^2^). Household income information was classified into quartiles: low, low-intermediate, intermediate-high, and high. Education levels were stratified into four categories: elementary school or lower, middle school, high school, college or higher. Alcohol intake was categorized into < 10 g/day and ≥ 10 g/day. Metabolic syndrome was defined for participants having three or more of the followings: (1) elevated waist circumference: ≥ 90 cm for men and ≥ 85 cm for women, (2) elevated triglycerides: ≥ 150 mg/dL or use of antidyslipidemic medications, (3) low high-density HDL-C: < 40 mg/dL for men,< 50 mg/dL for women, (4) elevated blood pressure: ≥ 130/85 mmHg or use of blood pressure lowering agents, (5) elevated fasting glucose: ≥ 100 mg/dL or on treatment for elevated glucose.

### Statistical analysis

Descriptive statistics for continuous variables are presented as median and interquartile range (IQR). Categorical variables are presented as numbers and proportions (%). Comparison of variables between groups was performed using Student’s t-test or Wilcox rank-sum test for continuous variables and Chi-square test for categorical variables. Generalized logistic regression was performed to determine whether the prevalence of NAFLD was different depending on physical activity after adjusting for potential confounding or mediating factors. When adjusting for age and sex, we used age per year as a continuous variable. In the fully adjusted model, we further adjusted for BMI (continuous), metabolic syndrome (yes vs. no), income levels (low, low-intermediate, intermediate-high, high), education levels (elementary or lower, middle school, high school, college or higher), smoking (current, ex-smoker, and never smoker), alcohol intake (< 10 g/day vs. ≥ 10 g/day), total physical activity (< 150 min/week vs. ≥ 150 min/week), and muscle strength exercise (< 2/week vs. ≥ 2/week). When specific domains of physical activity were assessed, other specific domains were adjusted. For recreation, travel (< 150 min/week vs. ≥ 150 min/week) and work activity (< 150 min/week vs. ≥ 150 min/week) were adjusted. For travel, recreation (< 150 min/week vs. ≥ 150 min/week) and work activity (< 150 min/week vs. ≥ 150 min/week) were adjusted. For work, recreation (< 150 min/week vs. ≥ 150 min/week) and travel activity (< 150 min/week vs. ≥ 150 min/week) were adjusted.

Subgroup analysis was performed to evaluate the relationship between physical activity or muscle strength exercise and NAFLD within each subgroup. Subgroups were predefined as follows: by age (< 65 years vs. ≥ 65 years), sex (male vs. female), BMI (< 25 kg/m^2^ vs. ≥ 25 kg/m^2^), metabolic syndrome (yes vs. no), muscle strength exercise (< 2/week vs. ≥ 2/week), and total physical activity (< 150 min/week vs. ≥ 150 min/week). All variables with a *p* value < 0.05 were considered statistically significant. All statistical analyses were performed using R version 3.6.3 (The R Foundation for Statistical Computing, Vienna, Austria).

## Results

Baseline characteristics of study participants are summarized in Table 1. Among 21,015 participants, 4942 (23.5%) had NAFLD. Participants with NAFLD were more likely to be older, male, current/ex-smokers, metabolically unhealthy, and have lower income, lower education, higher BMI than those without NAFLD. Participants with NAFLD also consisted of more participants who did not perform WHO recommended level (≥ 150 min/week) of physical activity and more participants who did not perform WHO recommended level (≥ 2/week) of muscle strength exercise (Table 2). When specific domain of physical activity was assessed, NAFLD participants were more likely to be inactive in recreation and travel domain activities, but not in work domain activity.

**Table 1:** Baseline characteristics of study participants by non-alcoholic fatty liver disease status (n = 21,015).

	Overall (n = 21,015)	NAFLD (+) (n = 4942)	NAFLD (−) (n = 16,073)	*p* value
Age (year)	51 (38–63)	52 (39–63)	50 (37–63)	< 0.001
Male	8659 (41.2)	2354 (47.6)	6305 (39.2)	< 0.001
Income^†^				
Low	4971 (23.7)	1283 (26.0)	3688 (22.9)	< 0.001
Low-intermediate	4963 (23.6)	1164 (23.6)	3799 (23.6)	
Intermediate-high	5343 (25.4)	1294 (26.2)	4049 (25.2)	
High	5686 (27.1)	1194 (24.2)	4492 (27.9)	
Education^†^				
Elementary or lower	4043 (19.2)	1151 (23.3)	2892 (18.0)	< 0.001
Middle school	2159 (10.3)	551 (11.1)	1608 (10.0)	
High school	6800 (32.4)	1590 (32.2)	5210 (32.4)	
College or higher	7987 (38.0)	1640 (33.2)	6347 (39.5)	
Occupation^†^	13,068 (62.2)	3056 (61.8)	10,012 (62.3)	0.3
Smoking				
Current smoker	3468 (16.5)	973 (19.7)	2495 (15.5)	< 0.001
Ex-smoker	4174 (19.9)	1025 (20.7)	3149 (19.6)	
Never smoker	13,373 (63.6)	2944 (59.6)	10,429 (64.9)	
Alcohol consumption				
< 10 g/day	13,025 (62.0)	3137 (63.5)	9888 (61.5)	0.014
≥ 10 g/day	7990 (38.0)	1805 (36.5)	6185 (38.5)	
Body mass index (kg/m^2^)	23.6 (21.4–25.9)	27.6 (26.0–29.6)	22.6 (20.8–24.4)	< 0.001
Metabolic syndrome*^†^	6359 (30.3)	3150 (63.7)	3209 (20.0)	< 0.001
Elevated waist circumference	5928 (28.2)	3618 (73.2)	2310 (14.4)	< 0.001
Elevated triglycerides	7571 (36.0)	2889 (58.4)	4682 (29.1)	< 0.001
Reduced HDL-C	7178 (34.2)	2402 (48.6)	4776 (29.7)	< 0.001
Elevated blood pressure	8131 (38.7)	2795 (56.6)	5336 (33.2)	< 0.001
Elevated fasting glucose	7309 (34.8)	2825 (57.2)	4484 (27.9)	< 0.001

Values are expressed as number (%) or median (quartile).

*NAFLD* non-alcoholic fatty liver disease, *HDL-C* high-density lipoprotein.

*Metabolic syndrome was defined when any three of five risk factors were present: elevated waist circumference: ≥ 90 cm for men, ≥ 85 cm for women; elevated triglycerides: ≥ 150 mg/dL or use of antidyslipidemic medications; reduced HDL-C: < 40 mg/dL for men, < 50 mg/dL for women; elevated blood pressure: ≥ 130/85 mmHg or use of antihypertensive medications; elevated fasting glucose: ≥ 100 mg/dL or use of antidiabetic medications.

^†^These variables had missing value. Number of participants with missing value were as follow: income (n = 52), education (n = 26), occupation (n = 12), metabolic syndrome (n = 64).

**Table 2:** Risk of non-alcoholic fatty liver disease according to the level of physical activity and muscle strength exercise.

	No. of subjects	NAFLD (%)	*p* value	Age and sex adjusted OR (95% CI)	Fully adjusted OR (95%CI)
Total physical activity (min/week)			< 0.001		
< 150	11,195	24.8		Ref	Ref
≥ 150	9820	22.1		0.86 (0.81–0.92)	0.86 (0.78–0.95)
Domains of physical activity					
Recreation (min/week)			< 0.001		
< 150	17,289	24.1		Ref	Ref
≥ 150	3726	20.7		0.79 (0.73–0.87)	0.77 (0.67–0.88)
Travel (min/week)			0.002		
< 150	14,608	24.1		Ref	Ref
≥ 150	6407	22.2		0.91 (0.85–0.98)	0.90 (0.81–1.01)
Work (min/week)			0.3		
< 150	19,678	23.4		Ref	Ref
≥ 150	1337	24.8		1.09 (0.95–1.25)	0.90 (0.73–1.10)
Muscle strength exercise (/week)			< 0.001		
< 2	16,732	24.6		Ref	Ref
≥ 2	4283	19.4		0.67 (0.62–0.73)	0.83 (0.73–0.94)

Fully adjusted model was adjusted for age (continuous), sex, body mass index (continuous), elevated waist circumference (yes vs. no), elevated triglycerides (yes vs. no), reduced high-density lipoprotein cholesterol (yes vs. no), diabetes mellitus (yes vs. no), hypertension (yes vs. no), income levels (Q1, Q2, Q3, and Q4), education (elementary or lower, middle school, high school, college or higher), smoking (current, ex-smoker, and never smoker), alcohol consumption (< 10 g/day vs. ≥ 10 g/day), total physical activity (< 150 min/week vs. ≥ 150 min/week), and muscle strength exercise (< 2/week vs. ≥ 2/week). For specific domains of physical activity, other domains were adjusted as follows: Recreation: travel and work activity (< 150 min/week vs. ≥ 150 min/week), Travel: recreation and work activity (< 150 min/week vs. ≥ 150 min/week), Work: recreation and travel activity (< 150 min/week vs. ≥ 150 min/week).

*NAFLD* non-alcoholic fatty liver disease, *OR* odds ratio, *CI* confidence interval.

**Figure 2:**
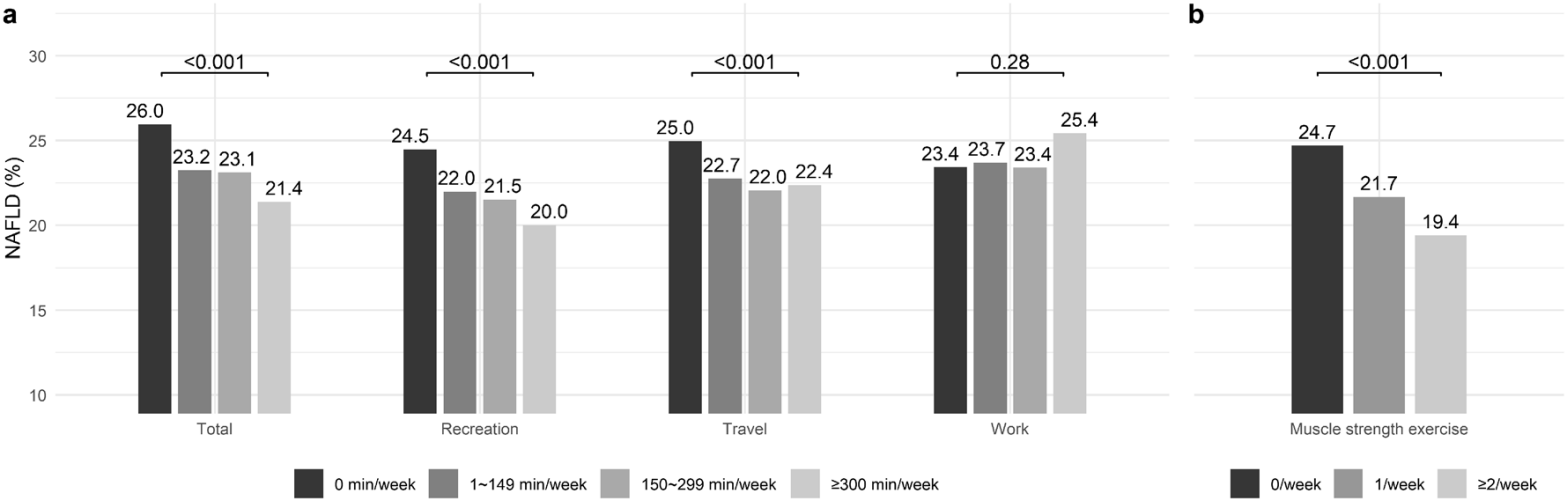
Prevalence of non-alcoholic fatty liver disease according to the level of (**A**) physical activity (total, recreation, travel, work) and (**B**) muscle strength exercise.

NAFLD prevalence was the highest among participants with 0 min/week of total physical activity. It was the lowest among those with ≥ 300 min/week of total physical activity (Fig. 2A). For the specific physical activity domain, NAFLD prevalence was the highest among participants with 0 min/week of recreation activity. It showed a dose-dependent decrease with an increase in recreation activity time. NAFLD prevalence was the highest among participants with 0 min/week of travel activity, showing no dose-dependent decrease with an increase in travel activity time. NAFLD prevalence was not different by work activity time. NAFLD was more prevalent in participants with 0 times of muscle strength exercise/week than in those with 1 time or ≥ 2 times of muscle strength exercise/week (Fig. 2B).

The fully adjusted odds ratio (OR) for NAFLD was 0.86 [95% confidence interval (CI): 0.78–0.95] when participants with ≥ 150 min/week of total physical activity were compared to those with < 150 min/week (Table 2). When the domain of physical activity was assessed separately, ≥ 150 min/week of recreation activity was negatively associated with the presence of NAFLD (OR: 0.77, 95% CI 0.67–0.88), whereas ≥ 150 min/week of travel activity and ≥ 150 min/week of work activity were not. The fully adjusted OR for NAFLD comparing participants with ≥ 2/week to those with < 2/week of muscle strength exercise was 0.83 (95% CI 0.73–0.94). When the level of physical activity was subdivided further, using 0 min/week activity as a reference group, ≥ 300 min/week of total physical activity, ≥ 300 min/week of recreation activity, ≥ 300 min/week of travel activity, 150–299 min/week of work activity, and ≥ 2/week of muscle strength exercise were negatively associated with the presence of NAFLD (Supplementary Table S1). The inverse association between the level of physical activity and NAFLD was dose-dependent for recreation and travel activities.

**Figure 3:**
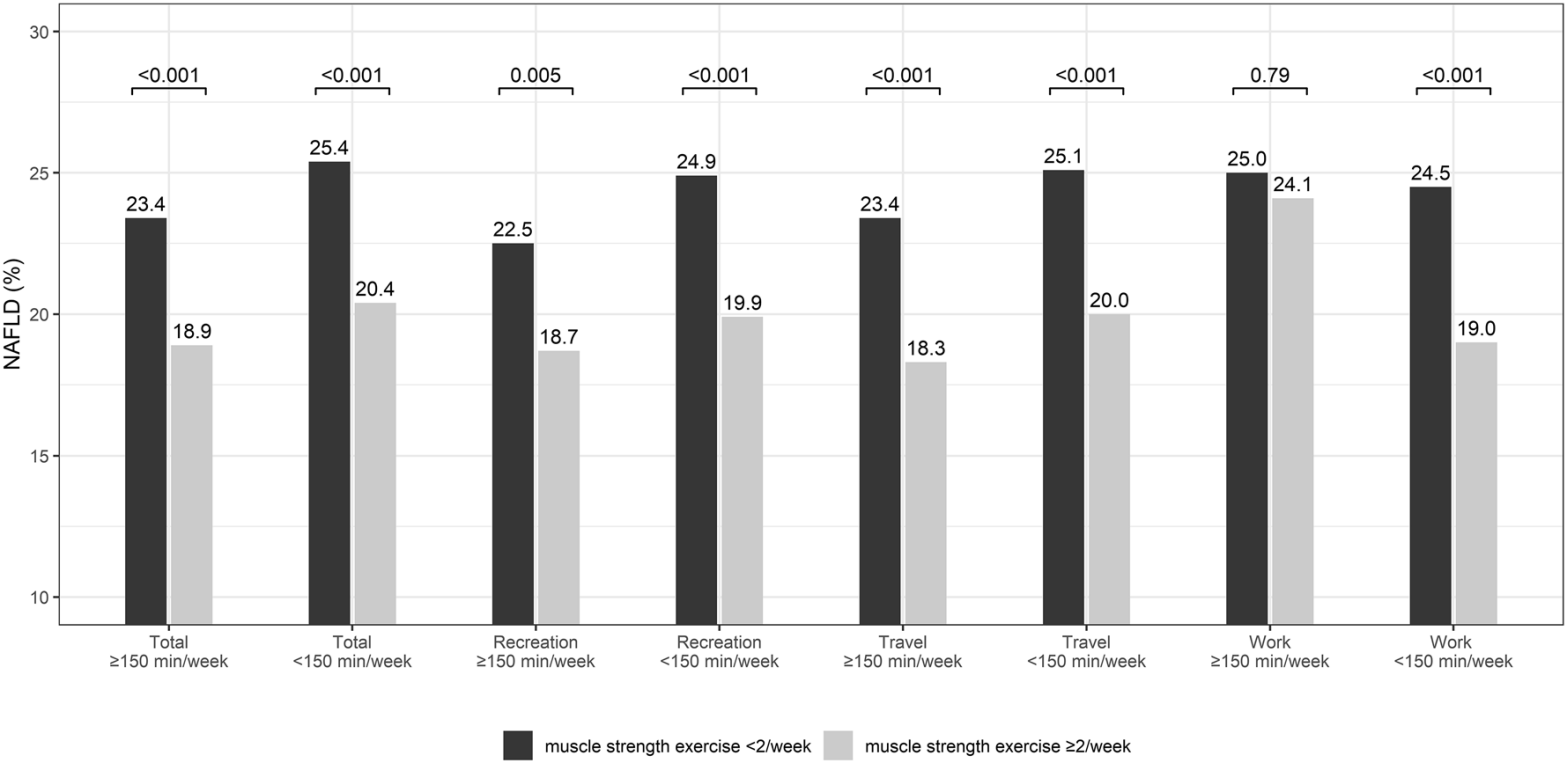
Prevalence of non-alcoholic fatty liver disease according to the level of muscle strength exercise (< 2/week and ≥ 2/week) in each physical activity group.

NAFLD prevalence was higher for participants with < 2/week of muscle strength exercise than those with ≥ 2/week for all levels of total and each specific domains of physical activity except for ≥ 150 min/week of work activity (Fig. 3).

**Figure 4:**
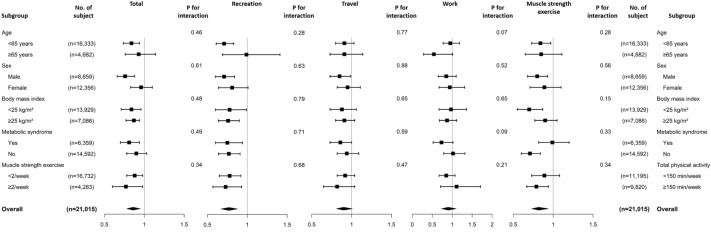
Odds ratio for the risk of NAFLD by the level of physical activity (< 150 min/week vs. ≥ 150 min/week) or muscle strength exercise (< 2/week vs. ≥ 2/week) in predefined subgroups. Models were adjusted for age (continuous), sex, body mass index (continuous), elevated waist circumference (yes vs. no), elevated triglycerides (yes vs. no), reduced high-density lipoprotein cholesterol (yes vs. no), diabetes mellitus (yes vs. no), hypertension (yes vs. no), income levels (Q1, Q2, Q3, and Q4), education (elementary or lower, middle school, high school, college or higher), smoking (current, ex-smoker, and never smoker), alcohol consumption (< 10 g/day vs.  10 g/day), total physical activity (< 150 min/week vs. ≥ 150 min/week), and muscle strength exercise (< 2/week vs. ≥ 2/week). For specific domains of physical activity, other domains were adjusted as follows: (1) For recreation activity, travel and work activities (< 150 vs. ≥ 150 min/week) were adjusted; (2) For travel activity, recreation and work activities (< 150 vs. ≥ 150 min/week) were adjusted; and (3) for work activity, recreation and travel activities (< 150 vs. ≥ 150 min/week) were adjusted.

In subgroup analysis, the association between physical activity or muscle strength exercise and NAFLD had no interaction in all predefined subgroups (Fig. 4).

## Discussion

In this nation-wide cross-sectional study, we found that WHO-recommended levels of total physical activity (≥ 150 min/week) and muscle strength exercise (≥ 2/week) were associated with a lower risk of NAFLD. When individual domains of physical activity were assessed, recreation activity (≥ 150 min/week), but not travel or work activity, was found to have a significant association with NAFLD. When the level of physical activity was subdivided further, ≥ 300 min/week of total physical activity, recreation activity, or travel activity, and 150–299 min/week of work activity were negatively associated with NAFLD. Muscle strength exercise ≥ 2/week showed a lower risk of NAFLD for all levels of total and each specific domains of physical activity except for ≥ 150 min/week of work activity. Subgroup analysis showed significant associations of NAFLD with total physical activity (≥ 150 min/week), recreation activity (≥ 150 min/week), and muscle strength exercise (≥ 2/week) in all predefined subgroups. These findings indicate that moderate-to-vigorous physical activity can reduce the risk of NAFLD. However, the impact varies by physical activity domains. Muscle strength exercise can also reduce the risk of NAFLD in most cases but except for ≥ 150 min/week of work activity.

In the present study, we demonstrated that the risk of NAFLD varied depending on the domains of physical activity. So far, most studies revealing the relationship between physical activity and NAFLD have focused on exercise intensity and recreation domain physical activity^22–25^. Only a few studies have examined effects of domains of physical activity on NAFLD^17,18^. In a cross-sectional study using NHANES, which assessed physical activity using GPAQ, work domain activity did not appear to be protective against NAFLD^17^. A population-based cohort study with 42,661 participants also showed that moderate to vigorous physical activity in the work domain had no discernible benefit on NAFLD^18^. In the present study, we observed that ≥ 150 min/week of recreation domain activity, ≥ 300 min/week of travel domain activity, and 150–299 min/week of work domain activity were associated with a reduced risk of NAFLD. Thus, in contrast to recreation or travel domain activity, vigorous level (≥ 300 min/week) of work domain activity might not be protective against NAFLD. Although the exact biological mechanism for these findings is unclear, the ‘physical activity paradox^14,26^, might partly explain it. At vigorous level, work physical activity is either excessively strenuous or excessively prolonged to be cardiorespiratory beneficial, resulting in persistently elevated blood pressure and heart rate. Furthermore, repeated actions without appropriate recuperation time increased the level of inflammation^14,27^. In addition, it has been shown that physical activity triggers beta-oxidation to promote damaged mitochondrial clearance^28^, but the anti-oxidative impact does not increase during intense occupational physical activity. ^29^ These findings indicate that physical activity should be differentiated by domains for health promotion effect. Especially, work activity should not be simply regarded as a substitute for recreation activity or as a measure of health-enhancing daily-life physical activity.

We also demonstrated that participants with ≥ 2/week of muscle strength exercise had a 17% lower risk of having NAFLD than those with < 2/week. Additionally, muscle strength exercise ≥ 2/week was associated with a reduced risk of NAFLD for all levels of total and each specific domains of physical activity except for ≥ 150 min/week of work activity. A previous randomized controlled trial comparing effects of resistance and aerobic training on NAFLD showed that both types of exercise were equally beneficial^30^. Muscle strength exercise might alter muscle properties by increasing glycolysis and decreasing insulin resistance, hence lowering hepatic steatosis^31^. According to a systematic review, muscle strength exercise consumes less energy than aerobic exercise to decrease steatosis^32^. Thus, muscle strength exercise could be a good option for individuals who lack motivation for aerobic exercise or who have limited cardiovascular fitness to do moderate-to-vigorous physical activity.

When interpreting our findings, certain restrictions must be acknowledged. Since it was a cross-sectional study, we could not infer the causal relationship between physical activity or muscle strength exercise and NAFLD. Furthermore, we used HSI to define NAFLD rather than biopsy or abdominal imaging, which might have resulted in a classification bias. Because data on physical activity were acquired based on remembrance, there might be a recall bias, which could lead to misclassification. However, since the health interview was conducted with the assistance of a trained surveyor, we were able to reduce nonresponse bias and measurement bias more effectively than other survey methods^33^. In addition, since oxidative stress or circulating endotoxin were not included in the KNHANES dataset, the pathophysiological mechanisms underlying the risk of NAFLD in relation to specific physical activity could not be investigated. Despite the aforementioned limitations, our study has a number of advantages. We utilized nation-wide representative data, which might have reduced selection bias. Also, we used a validated GPAQ collected by trained personnel to gather information on physical activity. Finally, with a large sample size, we could perform subgroup analyses with multiple risk factors adjustment.

In summary, increased physical activity and muscle strength exercise were associated with a reduced risk of NAFLD. However, the impact varied by the domain of physical activity. Moderate to vigorous recreation activity and vigorous travel activity were associated with reduced risk of NAFLD. However, work activity was associated with reduced risk of NAFLD only at a moderate level. Muscle strength exercise was associated with a reduced risk of NAFLD in most cases. These findings suggest that physical activity should be differentiated by domains for the management of NAFLD and that vigorous level of work activity might need to be avoided as a measure of health-enhancing daily-life physical activity. Also, muscle strength exercise could be a good option for individuals who could not perform moderate-to-vigorous physical activity.

## Supplementary Information

Supplementary Table S1.

## Data Availability

The data that support the findings of this study are available from the Korean National Health and Nutrition Examination Survey (KNHANES) but restrictions apply to the availability of these data, which were used under license for the current study, and so are not publicly available. Data are however available from the authors upon reasonable request and with permission of KNHANES.
