# Supplementary material for: Associations of physical activity domains and muscle strength exercise with non-alcoholic fatty liver disease: a nation-wide cohort study
Source: Sci Rep. 2023 Mar 23;13:4724. doi: 10.1038/s41598-023-31686-6 (PMC10036618; doi:10.1038/s41598-023-31686-6)
Supplement: Supplementary file 1 — Supplementary Table S1. [file 41598_2023_31686_MOESM1_ESM.docx]

**Table S1.** Risk of non-alcoholic fatty liver disease according to the subdivided level of physical activity and muscle strength exercise

|  | No. of  Subjects | NAFLD  (%) | *P* value | Age and sex adjusted OR (95% CI) | Fully adjusted OR (95%CI) |
| --- | --- | --- | --- | --- | --- |
| Total physical activity (min/week) |  |  | <0.001 |  |  |
| 0 | 6,261 | 26.0 |  | Ref | Ref |
| 1~149 | 4,934 | 23.2 |  | 0.89 (0.81-0.97) | 0.90 (0.79-1.03) |
| 150~299 | 4,180 | 23.1 |  | 0.88 (0.80-0.97) | 0.99 (0.86-1.13) |
| ≥300 | 5,640 | 21.4 |  | 0.77 (0.71-0.84) | 0.70 (0.62-0.81) |
| Domains of physical activity | | | | | |
| Recreation (min/week) |  |  | <0.001 |  |  |
| 0 | 14,918 | 24.5 |  | Ref | Ref |
| 1~149 | 2,371 | 22.0 |  | 0.86 (0.77-0.95) | 0.91 (0.78-1.07) |
| 150~299 | 1,692 | 21.5 |  | 0.83 (0.73-0.94) | 0.85 (0.70-1.02) |
| ≥300 | 2,034 | 20.0 |  | 0.73 (0.65-0.82) | 0.68 (0.57-0.81) |
| Travel (min/week) |  |  | <0.001 |  |  |
| 0 | 9,064 | 25.0 |  | Ref | Ref |
| 1~149 | 5,544 | 22.7 |  | 0.92 (0.85-0.99) | 0.94 (0.84-1.07) |
| 150~299 | 3,987 | 22.0 |  | 0.89 (0.81-0.97) | 0.92 (0.80-1.05) |
| ≥300 | 2,420 | 22.4 |  | 0.88 (0.79-0.98) | 0.83 (0.71-0.98) |
| Work (min/week) |  |  | 0.57 |  |  |
| 0 | 19,015 | 23.4 |  | Ref | Ref |
| 1~149 | 663 | 23.7 |  | 1.04 (0.86-1.25) | 0.86 (0.64-1.15) |
| 150~299 | 389 | 23.4 |  | 1.02 (0.80-1.29) | 0.63 (0.44-0.91) |
| ≥300 | 948 | 25.4 |  | 1.11 (0.96-1.30) | 1.04 (0.82-1.32) |
| Muscle strength exercise (/week) |  |  | <0.001 |  |  |
| 0 | 15,989 | 24.7 |  | Ref | Ref |
| 1 | 743 | 21.7 |  | 0.81 (0.67-0.97) | 1.01 (0.76-1.33) |
| ≥2 | 4,283 | 19.4 |  | 0.67 (0.62-0.73) | 0.83 (0.73-0.94) |

NAFLD, non-alcoholic fatty liver disease; OR: odds ratio; CI: confidence interval. Fully adjusted model was adjusted for age (continuous), sex, body mass index (continuous), elevated waist circumference (yes vs. no), elevated triglycerides (yes vs. no), reduced high-density lipoprotein cholesterol (yes vs. no), diabetes mellitus (yes vs. no), hypertension (yes vs. no), income levels (Q1, Q2, Q3, and Q4), education (elementary or lower, middle school, high school, college or higher), smoking (current, ex-smoker, and never smoker), alcohol consumption (<10 g/day vs. ≥10 g/day), total physical activity (<150 vs. ≥150 min/week), and muscle strength exercise (<2/week, ≥2/week). For specific domains of physical activity, other domains were adjusted as follows: Recreation: travel and work activity (<150 vs. ≥150 min/week), Travel: recreation and work activity (<150 vs. ≥150 min/week), Work: recreation and travel activity (<150 vs. ≥150 min/week).
